# Comparative Analysis of Outcomes and Influencing Factors in COVID-Associated Rhino-Orbital-Cerebral Mucormycosis: Globe Salvage Versus Exenteration

**DOI:** 10.7759/cureus.90777

**Published:** 2025-08-22

**Authors:** Vidhya Verma, Priti Singh, Saroj Gupta, Samendra Karkhur, Mahesh Verma

**Affiliations:** 1 Ophthalmology, All India Institute of Medical Sciences, Bhopal, Bhopal, IND; 2 Radiodiagnosis, Chirayu Medical College & Hospital, Bhopal, IND

**Keywords:** amphotericin b, covid-19, exenteration, globe salvage, mucormycosis, rocm

## Abstract

Aim

This study aimed to evaluate and compare the outcomes of globe-salvaging procedures versus orbital exenteration in patients with COVID-19-associated rhino-orbital-cerebral mucormycosis (ROCM) during the second wave of the pandemic in India.

Methods

An ambispective observational study was conducted at a tertiary care hospital in Central India between April and October 2021. Thirty-five consecutive patients with confirmed ROCM and recent COVID-19 infection were enrolled. Based on the extent of orbital involvement and surgical judgment, patients were categorized into either the Globe Salvage Group or the Exenteration Group. Clinical characteristics, comorbidities, treatment details, and outcomes were recorded, with a minimum follow-up of six months. The primary outcome was survival; secondary outcomes included disease recurrence, disease control, and postoperative complications. Statistical analyses were performed using IBM SPSS Statistics for Windows, Version 23.0 (Released 2015; IBM Corp., Armonk, NY, USA). This study adheres to the STrengthening the Reporting of OBservational studies in Epidemiology(STROBE) guidelines.

Results

The mean age of patients was 48.77 years, and 82.9% were male. Diabetes and prior corticosteroid use were present in 82.9% of patients. All cases were advanced ROCM (Stage III/IV). Of the 35 patients, 25 (71.4%) underwent globe-salvaging procedures, while 10 (28.6%) underwent orbital exenteration. Overall survival was 82.9%, with higher mortality in the Exenteration Group (40%) compared with the salvage group (8%). Disease recurrence occurred in 10% of the salvage group and 5% of the Exenteration Group.

Conclusions

When carefully selected, globe-salvaging procedures provide survival outcomes comparable to exenteration, with lower morbidity. Early diagnosis, control of risk factors, and timely intervention are essential to improving outcomes in ROCM.

## Introduction

The COVID-19 pandemic has led to numerous secondary health complications, one of the most devastating being the surge in cases of rhino-orbital-cerebral mucormycosis (ROCM). Mucormycosis is a rare opportunistic fungal infection caused by fungi of the order Mucorales, primarily affecting immunocompromised individuals. ROCM is a severe form of this infection, characterized by rapid progression from the nasal and paranasal sinuses to the orbit and CNS. Its pathophysiology is marked by aggressive angioinvasion, leading to vascular thrombosis, tissue necrosis, and high mortality if not treated promptly.

During the second wave of COVID-19 in India, a significant rise in ROCM cases was observed (0.14 cases per 1,000 population), particularly among patients with uncontrolled diabetes mellitus and those treated with corticosteroids or immunosuppressants for COVID-19 management [1,2]. Immune dysregulation induced by SARS-CoV-2, including cytokine storm, CD8+ T-cell exhaustion, and reduced T, B, and NK cell function, further predisposed susceptible individuals to invasive fungal infections such as ROCM.

Management of ROCM is challenging and typically requires a combination of antifungal therapy, surgical debridement, and control of underlying risk factors. One of the most debated aspects of surgical management is the choice between globe-salvaging procedures and orbital exenteration. Globe salvage surgery in ROCM involves aggressive sinus and orbital debridement, often combined with local amphotericin B instillation, with the aim of eradicating infection while preserving the eye. In contrast, orbital exenteration is a disfiguring but sometimes life-saving procedure that entails the removal of the entire orbital contents, including the globe, adnexa, and part of the periorbita, to control extensive disease. While exenteration may improve survival in advanced cases, it results in significant morbidity and disfigurement. Conversely, globe-salvaging approaches may preserve quality of life but raise concerns about residual fungal load and disease progression [2,3].

This study proposes that, in appropriately selected ROCM cases, early and aggressive globe-salvaging interventions can achieve disease control and survival outcomes comparable to those of orbital exenteration, without increasing mortality risk.

Despite numerous reports on COVID-associated ROCM, a specific gap remains in the literature regarding the comparative effectiveness of these two surgical approaches in terms of survival, disease control, and quality of life. This study addresses that gap by evaluating outcomes and influencing factors in patients who underwent either globe salvage or exenteration, aiming to provide evidence to guide clinical decision-making in the management of this life-threatening condition.

## Materials and methods

Study design and setting

An ambispective observational study was conducted at a tertiary care hospital in Central India during the second wave of the COVID-19 pandemic (April to October 2021). Thirty-five consecutive patients with a confirmed diagnosis of COVID-associated ROCM were included. The primary outcome was overall survival; secondary outcomes included disease recurrence, disease control, and postoperative outcomes.

Sample size estimation

The sample size of 35 was determined by the number of eligible patients with ROCM who presented to the study center during the designated timeframe. A formal power analysis was not performed because of the rare and outbreak-driven nature of the condition; this limitation is acknowledged. The study was exploratory in design, aiming to generate insights into outcome differences between globe-salvaging procedures and exenteration.

Ethical considerations

The study protocol was approved by the Institutional Research Review Board (IRRB) and the Institutional Human Ethics Committee (IHEC) (approval number IHEC-LOP/2021/IM0414). Written informed consent was obtained from all participants. The study adhered to the principles of the Declaration of Helsinki.

Inclusion and exclusion criteria

Patients were included if they had a confirmed diagnosis of ROCM established by microbiological methods (KOH mount, fungal culture, and/or histopathology) and a recent history of COVID-19 infection, defined as infection diagnosed within the previous six weeks by RT-PCR or antigen test. Exclusion criteria were incomplete medical records, absence of documented ROCM, or no confirmed COVID-19 infection.

Data collection

Data were collected from both electronic health records and physical charts. Extracted variables included demographics; details of COVID-19 infection (hospitalization, oxygen support, steroid use, vaccination status, and disease severity); comorbidities such as diabetes mellitus, hypertension, and other immunocompromising conditions (e.g., malignancy, transplant history, and immunosuppressive therapy); and the time interval between COVID-19 diagnosis, ROCM symptom onset, and treatment initiation. All data were de-identified prior to analysis to maintain confidentiality and comply with ethical standards.

Clinical evaluation

All patients underwent detailed ophthalmologic, ENT, and neurological evaluations by respective specialists. Standardized departmental protocols were followed, and whenever possible, the same evaluators were involved to minimize inter-observer variability.

Ophthalmologic evaluation included visual acuity, pupillary reflexes, ocular motility, proptosis measurement, fundus examination, and imaging correlation. Laboratory tests included complete blood count, renal and liver function tests, serum electrolytes, blood glucose levels, and HbA1c. Microbiological confirmation was obtained by KOH mount, fungal culture, and histopathological examination. The ENT evaluation consisted of nasal endoscopy, sinus imaging, and biopsy for microbiological confirmation. Neurological evaluation included cranial nerve examination and brain imaging (CT/MRI). All patients underwent contrast-enhanced MRI of the paranasal sinuses, orbits, and brain to assess disease extent. In cases of suspected bony involvement, contrast-enhanced CT was additionally performed. Orbital ultrasonography (B-scan) was not used, as MRI and CT provided more comprehensive evaluation of orbital and intracranial extension.

ROCM diagnosis and staging

ROCM diagnosis was confirmed through direct microscopy (KOH mount), fungal culture, and histopathology (evidence of angioinvasive fungal hyphae). Staging was performed according to the revised criteria by Honavar SG (2021): involvement of the nasal mucosa (Stage 1), orbit (Stage 2), and CNS (Stage 3). This staging system was used to guide treatment decisions.

Treatment protocols

All patients received antifungal therapy according to institutional guidelines. IV liposomal amphotericin B (5-10 mg/kg/day) was administered as the first-line agent, with isavuconazole or posaconazole used in cases of intolerance or contraindication. Antifungal therapy was administered intravenously for at least three to six weeks, followed by oral posaconazole or isavuconazole for consolidation, depending on disease severity and clinical response. Duration and dose adjustments were individualized based on renal function, disease burden, and treatment tolerance.

Prognostic indicators considered included baseline visual acuity, extent of orbital involvement, presence of intracranial extension, comorbidities (particularly uncontrolled diabetes), renal function status affecting amphotericin dosing, and timing of antifungal initiation after diagnosis.

Surgical management

Paranasal sinus debridement, endoscopic sinus surgery, or open resection was performed as indicated. The decision between globe salvage and orbital exenteration was based on imaging findings (primarily contrast-enhanced MRI, with contrast-enhanced CT additionally used in suspected bony involvement), extent of orbital involvement, visual potential, and systemic condition. No formal scoring system was used; decisions were reached by multidisciplinary consensus based on radiological and clinical parameters.

Patient grouping and follow-up

Patients were grouped into the Globe Salvage Group (managed with antifungal therapy and conservative or targeted surgical debridement) and the Exenteration Group (requiring removal of orbital contents due to extensive disease). Follow-up was conducted for a minimum of six months, assessing survival, disease progression, complications, vision status, and functional outcomes.

Statistical analysis

Data were analyzed using IBM SPSS Statistics for Windows, Version 23.0 (Released 2015; IBM Corp., Armonk, NY, USA). Continuous variables were expressed as mean ± SD or median with IQRs, depending on distribution. Categorical variables were expressed as frequencies and percentages. Normality was tested using the Shapiro-Wilk test. Independent t-tests or Mann-Whitney U tests were used for continuous variable comparisons, while chi-square or Fisher’s exact tests were applied for categorical data. P-values <0.05 were considered statistically significant. CIs were reported for key outcomes. Multivariate logistic regression was not performed because of the limited sample size, which is acknowledged as a study limitation.

This study was reported in accordance with the STrengthening the Reporting of OBservational studies in Epidemiology (STROBE) guidelines, ensuring standardized and transparent reporting of study design, participants, variables, data sources, statistical methods, and outcomes.

## Results

This ambispective study compared the clinical characteristics and treatment outcomes of COVID-19-associated ROCM in patients undergoing globe-salvaging procedures versus orbital exenteration, with a focus on factors influencing outcomes.

Demographics and clinical presentation

The mean age of the cohort was 48.77 ± 11.84 years, and the median age was 49.00 years (IQR: 40.00-57.50 years). The age range of the study population was 25-70 years, with a male predominance (82.9%). Diabetes mellitus was the most common comorbidity (82.9%), followed by hypertension (47.8%); 37.1% of patients had both diabetes mellitus and hypertension. None of the patients had received a COVID-19 vaccination. A majority (82.9%) had been treated with systemic corticosteroids during their COVID-19 illness. Most patients (94.3%) required hospitalization, and 77.1% required oxygen therapy. Detailed demographic and clinical characteristics are summarized in Table 1.

**Table 1:** Demographic and clinical characteristics of study population (n = 35)

Parameter	Category	Frequency (n = 35)	Percentage (%)	95% CI
Gender	Male	29	82.9%	-
	Female	6	17.1%	-
COVID-19 status	Active	15	42.9%	26.8-60.5%
	Recovered	20	57.1%	39.5-73.2%
Hospitalization during COVID-19	Yes	33	94.3%	79.5-99.0%
	No	2	5.7%	1.0-20.5%
Oxygen support during COVID-19	Yes	27	77.1%	59.4-89.0%
	No	8	22.9%	11.0-40.6%
Hyperglycemia status	Diabetes mellitus	29	82.9%	65.8-92.9%
	First-time hyperglycemia	6	17.1%	7.2-34.3%
Other comorbidity	Hypertension	22	47.8%	31.4-64.5%
Corticosteroid intake	Not taken	6	17.8%	7.2-34.3%
	Oral + IV steroid use	29	82.2%	65.7-92.8%

ROCM characteristics and contributing factors

ROCM was diagnosed in 20 patients (57.1%) who had recovered from COVID-19 and in 15 patients (42.9%) with active infection. The mean interval between COVID-19 diagnosis and ROCM onset was 27.85 days (range: 4-90 days). The mean cycle threshold (CT) value on RT-PCR was 15.2 ± 5.5, and the mean HRCT chest score was 11.8 ± 4.0. Most patients presented with Stage III ROCM (88.6%), while 11.4% had Stage IV disease with CNS involvement. Clinical features, including ocular findings, staging, sites of involvement, and surgical decisions, are summarized in Table 2.

**Table 2:** Management and outcome FESS, functional endoscopic sinus surgery; HM, hand motion vision; PNS, paranasal sinuses; ROCM, rhino-orbital-cerebral mucormycosis

Parameter	Frequency	Percentage	95% CI
Involved eye			
Unilateral	29	82.9%	65.7-92.8%
Bilateral	6	17.1%	7.2-34.3%
Involved eye/vision			
6/9-6/60	7	20.0%	9.1-37.5%
≤6/60	5	14.3%	5.4-31.0%
≤HM	23	65.7%	47.7-80.3%
Ocular findings			
Proptosis + partial ophthalmoplegia	29	82.9%	65.7-92.8%
Proptosis + total ophthalmoplegia	6	17.1%	7.2-34.3%
ROCM staging			
Stage 3	31	88.6%	72.3-96.3%
Stage 4	4	11.4%	3.7-27.7%
Area involved (CT scan)			
CNS involvement	4	11.4%	3.7-27.7%
PNS + orbit	31	88.6%	72.3-96.3%
Surgical management			
Exenteration	10	28.6%	13.6-43.5%
Globe salvage	25	71.4%	68.6-71.4%
FESS and sinus debridement	21	60.0%	42.2-75.6%
Admission outcome			
Mortality	6	17.1%	7.2-34.3%
Alive	29	82.9%	65.7-92.8%
Follow-up (six months)			
Stable	29	82.9%	65.7-92.8%
Expired	6	17.1%	7.2-34.3%

With respect to orbital involvement, 29 patients (82.9%) had unilateral disease, while six patients (17.1%) had bilateral disease. At presentation, vision status showed severe impairment (≤ hand movements) in 65.7% of eyes. A detailed comparison between the Globe Salvage and Exenteration Groups demonstrated statistically significant differences in ocular findings, disease staging, CNS involvement, and treatment approach. These comparisons are summarized in Table 3.

**Table 3:** Comparison of various parameters in Globe Salvage and Exenteration Group of COVID-19 associated ROCM ^*^ Significant at p < 0.05 FESS, functional endoscopic sinus surgery; HM, hand motion vision; PNS, paranasal sinuses; ROCM, rhino-orbital-cerebral mucormycosis

Parameter	Category	Globe Salvage (n = 26 eyes)	Globe Exenteration (n = 20 eyes)	p-Value
Involved eye	Right eye	13 (52.0%)	1 (10.0%)	<0.0013^*^
	Left eye	12 (48.0%)	3 (30.0%)	
	Both eyes	0 (0.0%)	6 (60.0%)	
Vision at presentation	6/9-6/60	7 (28.0%)	0 (0.0%)	0.0323^*^
	≤6/60	5 (20.0%)	0 (0.0%)	
	≤HM	13 (52.0%)	10 (100.0%)	
Ocular findings	Proptosis + partial ophthalmoplegia	25 (100.0%)	4 (40.0%)	<0.0013^*^
	Proptosis + total ophthalmoplegia	0 (0.0%)	6 (60.0%)	
ROCM staging	Stage 1	0 (0.0%)	0 (0.0%)	0.0043^*^
	Stage 2	0 (0.0%)	0 (0.0%)	
	Stage 3	25 (100.0%)	6 (60.0%)	
	Stage 4	0 (0.0%)	4 (40.0%)	
Area involved in the CT scan	CNS involvement	0 (0.0%)	4 (40.0%)	0.0043^*^
	PNS + orbit	25 (100.0%)	6 (60.0%)	
Medical treatment	Liposomal amphotericin B	25 (100.0%)	10 (100.0%)	1.0002
	Other	0 (0.0%)	0 (0.0%)	
Surgical treatment	No intervention	4 (16.0%)	0 (0.0%)	<0.0013^*^
	Exenteration	0 (0.0%)	10 (100.0%)	
	FESS + sinus debridement	21 (84.0%)	0 (0.0%)	

Significant differences were also observed between the active and recovered COVID-19 groups. The mean age was higher in the active group (54.33 ± 12.26 years) compared with the recovered group (44.60 ± 9.98 years; p < 0.05). Corticosteroid use was more frequent in the active group (92.6%) than in the recovered group (75%). New-onset hyperglycemia was observed in some patients who had not received corticosteroids, suggesting a possible independent metabolic effect of COVID-19.

Management

Of the 35 patients, 21 (60.0%) underwent functional endoscopic sinus surgery (FESS) or paranasal sinus (PNS) debridement. Orbital exenteration was performed in 10 patients (28.6%), while 4 patients (11.4%) did not undergo any surgical intervention. Exenteration was primarily reserved for patients with bilateral orbital disease, Stage IV involvement, or CNS extension. Given the advanced stage of ROCM at presentation, all exenterations were lid-involving. The mean time from symptom onset to exenteration was 28.9 ± 14.6 days.

RRs and ORs were calculated for predictors such as CNS involvement, total ophthalmoplegia, and advanced staging to identify factors associated with exenteration and mortality. These findings are summarized in Table 4.

**Table 4:** Association of various attributing factors and outcomes of ROCM associated with COVID-19 ROCM, rhino-orbital-cerebral mucormycosis

Predictor/risk factor	Outcome	OR (95% CI)	RR (95% CI)
COVID-19 status: active	Globe salvage	2.15 (0.45-10.29)	1.23 (0.78-1.93)
COVID-19 status: active	Globe exenteration	0.46 (0.10-2.22)	0.57 (0.18-1.67)
COVID-19 status: recovered	Globe salvage	0.46 (0.10-2.22)	0.81 (0.52-1.28)
COVID-19 status: recovered	Globe exenteration	2.15 (0.45-10.29)	1.75 (0.60-5.60)
Ocular findings: proptosis + total ophthalmoplegia	Globe exenteration	73.67 (3.50-1549.31)	7.25 (3.12-18.19)
ROCM staging: Stage 4	Globe exenteration	35.31 (1.68-742.57)	5.17 (2.14-10.88)
Area involved (CT scan): CNS involvement	Globe exenteration	35.31 (1.68-742.57)	5.17 (2.14-10.88)
Ocular outcome: globe salvage	Admission outcome: alive	7.67 (1.12-52.32)	1.53 (1.05-2.96)
Ocular outcome: globe exenteration	Admission outcome: death	7.67 (1.12-52.32)	5.00 (1.21-20.69)

All patients (100%) received IV liposomal amphotericin B. Posaconazole or isavuconazole was administered in selected cases where amphotericin B was not tolerated. Three patients (8.6%) could not tolerate amphotericin B due to renal dysfunction or adverse effects and were switched to oral posaconazole or isavuconazole.

Of the total cohort, 25 patients (71.4%) were managed with globe-salvaging procedures, while 10 patients (28.6%) underwent orbital exenteration. Baseline disease severity was significantly higher in the Exenteration Group (Stage IV or CNS involvement). Uncontrolled diabetes was more common in the Exenteration Group (60%) compared with the Globe Salvage Group (35%). Notably, the RR of requiring exenteration was 1.35 times higher in recovered patients compared with those with active COVID-19.

**Figure 1:**
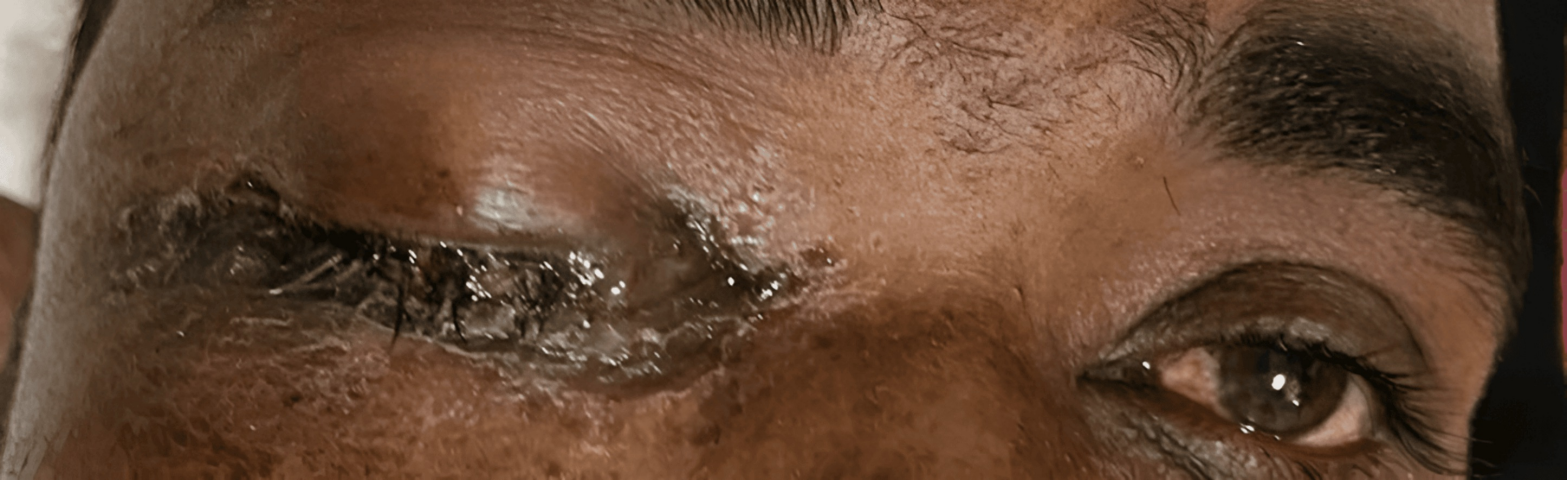
Preoperative image of a patient in the Globe Salvage Group Clinical photograph showing periocular swelling, ptosis, and black eschar at the medial canthus in a patient with COVID-associated rhino-orbital mucormycosis.

Patients in the Globe Salvage Group typically presented with periocular swelling, ptosis, and localized eschar formation (Figure 1).

**Figure 2:**
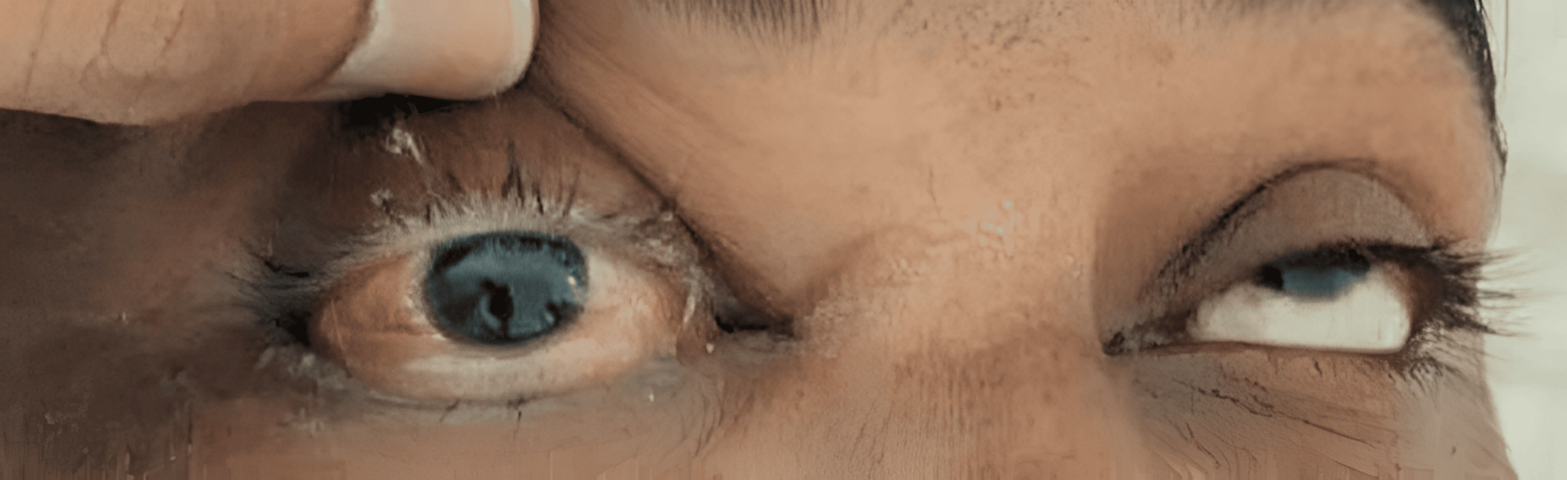
Posttreatment image of a patient in the Globe Salvage Group Postoperative photograph after endoscopic debridement and retrobulbar amphotericin B injection, demonstrating resolution of periocular edema with preservation of the globe at follow-up.

Posttreatment imaging of the same patient shows resolution of edema with preservation of the globe following endoscopic debridement and retrobulbar amphotericin B injections (Figure 2).

**Figure 3:**
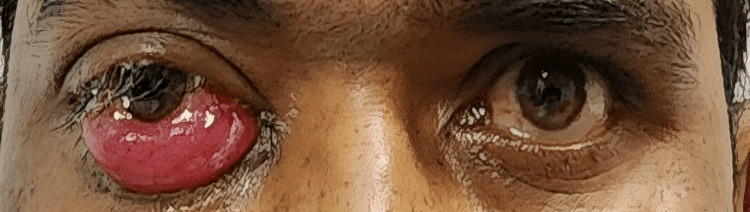
Preoperative image of a patient in the Globe Salvage Group Clinical photograph showing periocular swelling, conjunctival chemosis, and black eschar in a patient with COVID-associated rhino-orbital mucormycosis.

Another patient presented with periocular swelling, chemosis, and localized eschar formation (Figure 3).

**Figure 4:**
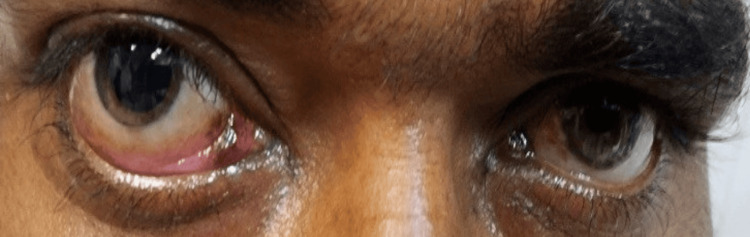
Posttreatment image of a patient in the Globe Salvage Group Postoperative photograph of the same patient after endoscopic debridement and retrobulbar amphotericin B injection, demonstrating resolution of periocular edema with preservation of the globe at follow-up.

Posttreatment photograph of the same patient demonstrates resolution of edema with preservation of the globe after endoscopic debridement and retrobulbar amphotericin B injections (Figure 4).

In contrast, patients in the Exenteration Group often showed extensive orbital involvement with severe proptosis, ophthalmoplegia, and loss of vision. Post-exenteration follow-up patients presented with well-healed sockets with healthy granulation tissue.

Follow-up outcomes (six months)

At six-month follow-up, the overall survival rate was 82.9% (29/35 patients). Mortality was higher in the Exenteration Group (40%) compared to the Globe Salvage Group (8%). Additionally, patients with active COVID-19 at ROCM diagnosis had a 1.76-fold higher mortality risk. Disease recurrence occurred in 10% of patients in the Globe Salvage Group and 5% in the Exenteration Group, though the difference was not statistically significant.

In the salvage group, vision could be preserved to a functional level (≥ perception of light) in 52% of eyes, highlighting the benefit of early intervention. In the Exenteration Group, although vision was lost, a healthy socket was achieved in all cases at follow-up, allowing for eventual prosthetic rehabilitation.

## Discussion

ROCM emerged as a significant secondary infection during the COVID-19 pandemic, particularly among patients with uncontrolled diabetes and those receiving systemic corticosteroids. Singh et al. documented a dramatic increase in mucormycosis cases in India during the pandemic, highlighting its strong association with COVID-19 and its management practices [2]. Our study adds to the growing body of literature by analyzing clinical parameters and outcomes in post-COVID-19 ROCM, focusing on the comparative effectiveness of globe-salvaging strategies versus orbital exenteration.

Demographics and risk factors

The mean age of patients was 48.77 years, with a marked male predominance (82.9%). This aligns with previous reports suggesting higher susceptibility among middle-aged males with comorbidities [3]. The potential hormonal or behavioral differences contributing to increased risk in males, including delayed health-seeking behavior or higher incidence of diabetes, warrant further exploration. Diabetes mellitus was the most common comorbidity, present in 82.9% of patients, consistent with previous findings that hyperglycemia predisposes individuals to mucormycosis by impairing neutrophil function and altering iron metabolism [4,5]. Additionally, 82% of patients received corticosteroids during COVID-19 treatment, known to exacerbate hyperglycemia and suppress immunity, further increasing fungal susceptibility [6-8].

While the study reports a marked male predominance (29 males versus six females), consistent with existing literature suggesting higher susceptibility in males, sex-specific analyses were limited due to the small number of female participants [9]. Descriptively, among the six female patients, four underwent globe-salvaging procedures, while two required exenteration. In contrast, among the 29 male patients, 21 underwent globe salvage and 8 underwent exenteration. Mortality occurred in one female and five male patients. However, due to the limited sample size, especially in the female subgroup, meaningful statistical comparisons could not be performed.

COVID-19 and ROCM association

The mean interval from COVID-19 diagnosis to ROCM onset was 27.85 days, indicating a delayed fungal manifestation post-viral infection. ROCM was notably more frequent in patients with severe COVID-19 illness (94.28%) and those who required oxygen therapy (77.14%), consistent with the hypothesis that hypoxia and endothelial damage facilitate fungal invasion [10]. A lower mean CT value (15.2 ± 5.5) and moderate HRCT chest score (11.8 ± 4) in our cohort reflect high viral load and associated pulmonary compromise, potentially contributing to disease severity [11].

Disease severity and surgical interventions

Most patients presented with advanced-stage disease: Stage III in 88.57% and Stage IV in 11.42%. Orbital exenteration was performed in 28.57% of cases and was significantly associated with bilateral orbital involvement, CNS extension, and severe disease (RR = 7.2). These findings echo the conclusions of Hoenigl et al., who emphasized early aggressive intervention in preventing intracranial spread and improving outcomes [11].

Globe salvage versus exenteration outcomes

Globe salvage was achieved in 71.4% of patients, while 28.6% required exenteration. The mortality rate was significantly higher in the Exenteration Group (40%) compared to 8% in the Globe Salvage Group, underlining the prognostic implications of disease severity at presentation [12,13]. Although exenteration may reduce intracranial extension in advanced cases, it carries a substantial physical and psychological burden. Disease recurrence was noted in 10% of the Globe Salvage Group and 5% of the Exenteration Group, though this difference was not statistically significant.

Patients in the Exenteration Group had a higher frequency of uncontrolled diabetes (60%) compared to the Globe Salvage Group (35%), underscoring the critical role of glycemic control in determining treatment outcomes.

Medical and surgical management

All patients received liposomal amphotericin B, consistent with current guidelines for invasive mucormycosis [14]. Endoscopic sinus surgery and debridement were performed in 60% of cases, while exenteration was reserved for those with extensive orbital or CNS involvement, following a stepwise, severity-based surgical protocol [15].

Survival and mortality analysis

Overall mortality was 17.1%. Active COVID-19 cases had a 1.76 times higher mortality than recovered patients, highlighting the additional immunological burden posed by active viral infection. At six-month follow-up, 82.9% of patients had survived, reinforcing the importance of early diagnosis and multidisciplinary intervention [16].

Impact of vaccination

None of the patients in our study was vaccinated against COVID-19, which may have contributed to the high incidence and severity of ROCM. Recent evidence supports that vaccination reduces the severity of COVID-19 and the likelihood of secondary infections like mucormycosis [17]. This finding highlights the need for proactive vaccination in high-risk groups, including diabetics and the immunocompromised..

Strengths of the study

One of the major strengths of this study is its focused comparison of globe-salvaging strategies versus exenteration in a well-characterized cohort of ROCM patients. The six-month follow-up provides meaningful insight into both short- and medium-term outcomes, including survival, recurrence, and complications. The study also highlights the role of systemic factors, such as diabetes and COVID-19 severity, in influencing the need for radical surgical intervention. Being conducted in a high-burden region during the peak of the COVID-19 pandemic, it reflects real-world challenges and decision-making in resource-constrained settings.

Limitations

Given the rarity of ROCM outbreaks, particularly in the context of COVID-19, this study offers valuable insights despite certain inherent limitations. Conducted at a single tertiary care center with a modest sample size (n = 35), the findings may not be widely generalizable but nonetheless contribute meaningfully to the limited literature on this uncommon condition. The retrospective design, while appropriate for rapid analysis during a pandemic, may introduce selection bias and limit control over potential confounders. Additionally, the small number of female participants precluded sex-stratified analysis, and psychosocial outcomes following disfiguring procedures such as exenteration were not assessed. The absence of a standardized surgical decision protocol, with procedures tailored to individual disease extent and patient factors, is also a limitation of this study. Future prospective multicentric studies with larger and more balanced cohorts are encouraged to build on these findings, particularly exploring sex-related variations in clinical presentation, management strategies, and long-term outcomes of ROCM.

## Conclusions

This study underscores the complex interplay between COVID-19 severity, underlying comorbidities, particularly diabetes, and the type of surgical intervention in shaping outcomes of post-COVID-19 ROCM. The markedly higher mortality observed in the Exenteration Group reflects the aggressive course of advanced-stage disease and emphasizes the critical need for early diagnosis and timely, globe-salvaging interventions when feasible. Effective glycemic control, rational corticosteroid use, and proactive COVID-19 vaccination are essential strategies to reduce ROCM incidence and improve patient survival.

While findings provide valuable clinical insight, the single-center, retrospective design and limited sample size constrain the generalizability of results. Future prospective multicentric studies with longer follow-up are needed to validate these observations and to explore long-term functional and psychosocial outcomes. Optimizing treatment algorithms tailored to disease stage and systemic health remains a key direction for further research.
